# Investigations on Thermal Transitions in PDPP4T/PCPDTBT/AuNPs Composite Films Using Variable Temperature Ellipsometry

**DOI:** 10.3390/polym17050704

**Published:** 2025-03-06

**Authors:** Paweł Jarka, Barbara Hajduk, Pallavi Kumari, Henryk Janeczek, Marcin Godzierz, Yao Mawuena Tsekpo, Tomasz Tański

**Affiliations:** 1 Department of Engineering Materials and Biomaterials, Silesian University of Technology, 18a Konarskiego Str., 41-100 Gliwice, Poland; pawel.jarka@polsl.pl (P.J.); yao.tsekpo@polsl.pl (Y.M.T.);; 2 Centre of Polymer and Carbon Materials, Polish Academy of Sciences, 34 Marie Curie-Skłodowska Str., 41-819 Zabrze, Poland; pkumari@cmpw-pan.pl (P.K.); hjaneczek@cmpw-pan.pl (H.J.); mgodzierz@cmpw-pan.pl (M.G.)

**Keywords:** variable temperature ellipsometry, thermal transitions, polymer/nanoparticle composites, thin films

## Abstract

Herein, we report a comprehensive investigation on the thermal transitions of thin films of poly [2,5-bis(2-octyldodecyl)pyrrolo[3,4-c]pyrrole-1,4(2H,5H)-dione -3,6-diyl)-alt-(2,2′;5′,2″;5″,2′″-quaterthiophen-5,5′″-diyl)]PDPP4T, poly[2,6-(4,4-bis-(2-ethy-lhexyl)-4H-cyclopenta [2,1-b;3,4-b′]dithiophene)-alt-4,7(2,1,3-benzothiadiazole)] PCPDTBT, 1:1 blend of PDPP4T and PCPDTBT, and their composites with gold nanoparticles (AuNPs). The thermal transitions of these materials were studied using variable temperature spectroscopic ellipsometry (VTSE), with differential scanning calorimetry (DSC) serving as the reference method. Based on obtained VTSE results, for the first time, we have determined the phase diagrams of PDPP4T/PCPDTBT and their AuNPs composites. The VTSE measurements revealed distinct thermal transitions in the thin films, including characteristic temperatures corresponding to the pure phases of PDPP4T and PCPDTBT within their blends. These transitions were markedly different in the AuNPs composites compared to the neat materials, highlighting the unique interactions between the polymer matrix and AuNPs. Additionally, we explored the optical properties, surface morphology, and crystallinity of the materials. We hypothesize that the observed variations in thermal transitions, as well as the improvement in optical properties and crystallinity, are likely influenced by localized surface plasmon resonance (LSPR) and passivation phenomena induced by the AuNPs in the composite films. These findings could have important implications for the design and optimization of materials for optoelectronic applications.

## 1. Introduction

The widespread integration of semiconducting organic polymers into the organic electronics industry has yet to be fully realized. However, due to their low cost and high efficiency, these materials have been proven to be a promising candidate for future renewable energy sources (RESs). One of the most critical challenges currently being addressed by numerous scientific research groups is the thermal stability of these materials. The study of thermal transitions in organic semiconductor materials is one of the essential factor for optimizing the performance and stability of organic electronic devices. Addressing this issue requires comprehensive studies of their morphology (both surface and internal), structural properties, and the impact of fabrication parameters [1,2], which influence the chemical and physical structure of active layers in devices, such as organic light-emitting diodes (OLEDs) [3,4] and organic field-effect transistors (OFETs) [5,6]. These studies typically focus on factors such as polymer molecular weight, thin-film deposition conditions, solvent selection, and variations in the composition of donor-acceptor polymer blends, all of which significantly affect charge transport and the overall performance of electronic devices [7,8,9,10,11]. A major research direction involves exploring polymer with inorganic metal oxides and metal nanoparticle (NP) composites for enhancing the electronic and optical properties of polymers [12,13], which are widely utilized across multiple industries [14,15,16,17,18]. In the field of organic electronics, the most commonly used inorganic metal nanoparticles are gold and silver nanoparticles [19,20]. These metal nanoparticles enhance light absorption primarily through the effect of their surface plasmon resonance (SPR). When exposed to light, the nanoparticles support the collective oscillations of conduction electrons at specific frequencies that resonate with the incident light. This phenomenon significantly amplifies the light absorption, with the degree of enhancement depending on the size and morphology of the nanoparticles. For instance, Pillai et al. demonstrated a 33% increase in photocurrent for silicon solar cells based on thin films by incorporating metallic nanoparticles. This improvement results from the enhanced absorption or scattering of light, driven by the size-dependent properties of the particles, which enable more efficient light harvesting [21]. Gold nanoparticles (AuNPs), in particular, are a highly effective tool in manipulating light–matter interactions within organic solar cell (OSC) architectures. AuNPs can be used in both single and mixed morphologies, providing plasmonic enhancement and light trapping properties. The unique SPR characteristics of AuNPs allow for the manipulation and control of light at the nanoscale, optimizing the interaction between light and the material [22].

To improve the thermal and electrical characteristics of organic electronics, one potential research area in the accounts is the study of composite films that combine semiconducting organic polymers with inorganic nanoparticles, namely gold nanoparticles (AuNPs). One such promising composite system is based on two semiconducting polymers, PDPP4T and PCPDTBT, which are known for their small optical band gaps and high charge carrier mobility, making them ideal candidates for organic solar cells [23,24]. In PDPP4T, the diketopyrrolopyrrole (DPP) unit enhances electron affinity, charge transport, and π-π stacking, while the thiophene segments improve backbone conjugation and charge mobility. Similarly, PCPDTBT incorporates cyclopenta[2,1-b;3,4-b′]dithiophene (CPDT) for high charge mobility and strong π-π interactions, while benzothiadiazole (BT) boosts electron affinity and charge separation. The alkyl side chains of both polymers improve solubility and processability making it well-suited for solution-based fabrication [25]. Incorporating gold nanoparticles (AuNPs) into blends of these polymers offers a unique opportunity to study how nanoparticles influence thermal transitions in these materials, ultimately affecting the material properties and device performance.

In this study, we utilize variable temperature ellipsometry to investigate the thermal transitions in PDPP4T/PCPDTBT/AuNPs composite films. This powerful technique enables the precise measurement of the film’s optical properties as a function of temperature, providing valuable insights into the behavior of the composite films. Key thermal transitions, such as the glass transition temperature (*T_g_*), cold crystallization temperature (*T_cc_*), and melting temperature (*T_m_*), are critical for understanding the stability and operational limits of organic materials in electronic applications [26,27,28,29,30]. By studying these transitions, we aim to uncover how the incorporation of AuNPs affects the thermal properties of the polymers and polymer composites and explore potential pathways for improving the performance and longevity of organic electronic devices like organic solar cells and light-emitting diodes.

A significant innovation of this work is the development of a phase diagram for the PDPP4T/PCPDTBT blend, created for the first time using variable temperature spectroscopic ellipsometry, with differential scanning calorimetry (DSC) serving as the reference technique [26,27]. This approach builds upon previous studies where thermal transitions were determined using raw ellipsometric data (temperature-induced changes in ellipsometric angles *Ψ* and *Δ* at a selected wavelength). Along the thermal transition, we also studied the optical properties and surface morphology. Our results indicate that incorporating AuNPs significantly impacts the thermal stability and crystallization behavior of the material, which is crucial for applications in organic field-effect transistors (OFETs). This work presents a novel contribution to the field, shedding light on the detail investigation on thermal transition of PDPP4T/PCPDTBT/AuNPs composites and their potential for use in organic electronics.

## 2. Experimental

### 2.1. Materials

**Figure 1:**
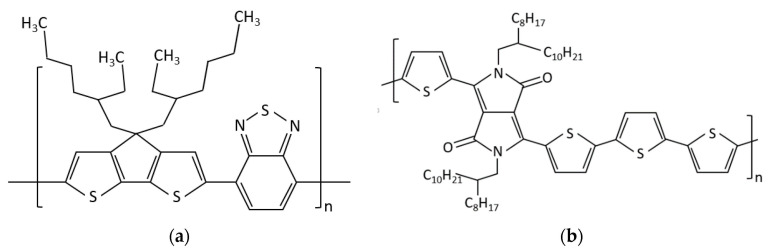
Chemical structures of PCPDTBT (**a**) and PDPP4T (**b**).

We have used two commercial, semiconducting polymers, poly[2,6-(4,4-bis-(2-ethy-lhexyl)-4H-cyclopenta [2,1-b;3,4-b′]dithiophene)-alt-4,7(2,1,3-benzothiadiazole)]M2096A3—PCPDTBT (molar mass M_w_ = 66.499 kDa) [31,32,33] and poly[2,5-bis(2-octyldodecyl)pyrrolo[3,4-c]pyrrole-1,4(2H,5H)-dione -3,6-diyl)-alt-(2,2′;5′,2″;5″,2′″-qua-terthiophen-5,5′″-diyl)] M333—PDPP4T (molar mass M_w_ = 84 kDa) [25,34,35], which were supplied by Ossila (Ossila Ltd., Sheffield, UK). The chemical structures of these polymers are presented in Figure 1a,b. The gold nanoparticles—AuNPs with a 20 nm diameter were supplied by Sigma-Aldrich (Merck KGaA, Darmstadt, Germany).

### 2.2. Samples Preparation

The polymers PCPDTBT and PDPP4T were dissolved in chloroform at a concentration of 10 mg/mL. Solutions containing AuNPs were prepared with a 10% weight concentration of nanoparticles, while maintaining a constant overall concentration of 10 mg/mL. The specific weight concentrations are provided in Table 1 and Table 2.

All solutions were placed in an ultrasonic bath at 40 °C for approximately 30 min. The films were prepared using spin-coating and casting techniques onto silicon substrates (covered with 90 nm of SiO_2_) and microscope slides (0.1 mm thick). The casting technique was used for the very thick samples intended for DSC measurements, where the material was scratched from the surface. The thickness of the prepared films was determined ellipsometrically and is presented in Table 3, below.

The substrates were cleaned following a standard procedure, which included rinsing with deionized water, ultrasonic cleaning with organic solvents (acetone and methanol), and drying in a centrifuge. The prepared films were annealed on a hot plate for 10 min at 120 °C to remove any residual solvent, then stored at room temperature in a dry laboratory box. The films were kept in a rubber-sealed storage container, partially filled with a hygroscopic gel, and stored in a nitrogen glove box. The films deposited onto silicon substrates were used for ellipsometric. In case of films deposited onto microscopic glass substrates, the prepared samples were used for XRD and microscopic analysis.

### 2.3. Methods

Ellipsometric measurements were conducted using a SENTECH SE 850E ellipsometer (Sentech, Krailling, Germany), operated with SpectraRay 3 software, covering a spectral range of 240–2500 nm. Temperature control during measurements was achieved using a temperature chamber under low vacuum conditions (10^−1^ Tr). The temperature was regulated by an INSTEC mK 1000 controller (Instec, Boulder, CO, USA), utilizing an electrical heater and a liquid nitrogen circuit. The optical windows of the vacuum chamber allowed for measurements at a 70° incidence angle. A standard temperature protocol was applied, where each sample was heated to 300 °C for 2 min and then rapidly cooled to −100 °C. Temperature curves were recorded during the heating cycles at a rate of 2 °C/min.

Differential scanning calorimetry (DSC) measurements were performed using a DSC Q2000 (TA Instruments, Newcastle, DE, USA) with aluminum sample pans. Thermal characteristics were assessed under a nitrogen atmosphere with a gas flow rate of 50 mL/min. The instrument was calibrated using high-purity indium standards, and the heating and cooling rates were set to 20 °C/min. DSC measurements were taken for the powders of PDPP4T and PCPDTBT, as well as for their 50% blend, which were scratched from the glass substrate.

X-ray diffraction (XRD) scans were carried out on polymer films deposited on Si/SiO_2_ substrates using a D8 Advance diffractometer (Bruker, Karlsruhe, Germany) with a Cu-Kα cathode (λ = 1.54 Å) in coupled Two-Theta/Theta mode. The scan rate was 1.2°/min with a step size of 0.02° in the 2θ range of 2° to 60° (dwell time 1 s). Background subtraction, accounting for air scattering, was performed using the DIFFRAC.EVA software V5.1.

The surface morphology of the prepared thin films, including pure materials, their blends, and composites, was analyzed using an Park Systems XE 100 atomic force microscope, operated by XEI computer program (Park Systems, Suwon, Republic of Korea), operating in contact mode in air with a constant force regime.

## 3. Results and Discussion

### 3.1. Optical Analysis

Spectroscopic ellipsometry is a reflection optical technique that examines the change in polarization parameters of light reflected from the surface of a sample. Light incident on the sample is linearly polarized, while after reflection, it is elliptically polarized. The two main parameters measured by the ellipsometer are called ellipsometric angles—*Ψ* and *Δ*. The first parameter, *Ψ*, is the change of the polarization amplitude, while *Δ* is the phase shift between the electrical vectors −p and −s of the electromagnetic beam before and after reflection from the sample. The relation that connects these parameters is called the main equation of ellipsometry, describing the complex reflectance coefficient ρ.

(1) $$\rho =e^{i\Delta }tan\Psi$$

**Scheme 1:**
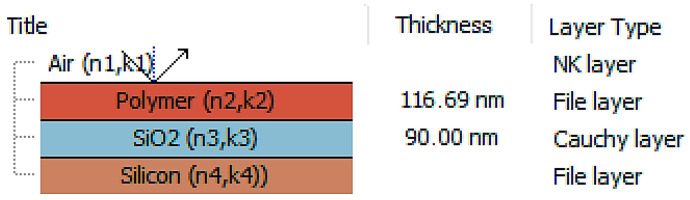
The ellipsometric model used for the measurement fittings.

Measurements of ellipsometric angles *Ψ* and *Δ* were performed in the variable angle mode (VASE) of the ellipsometer, in the range of angles 40–70°, every 10°, at room temperature, in the wavelength range 240–930 nm. The absorption spectra were derived using an ellipsometric model consisting of four layers, as depicted in Scheme 1. The layers representing air, polymer, SiO_2_, and silicon were treated as film-type layers, where the modulation was achieved by fitting the refractive index (*n*) and extinction coefficient (*k*) at each wavelength (λ). It is important to note that these parameters were fitted as pairs, with each value of n and k corresponding to the same λ. The silicon oxide layer was modelled using a Cauchy layer, with the corresponding relationship detailed in our previous works [26,27]. The mean square error (MSE) for the fitting results was approximately MSE ≈ 0.7.

**Figure 2:**
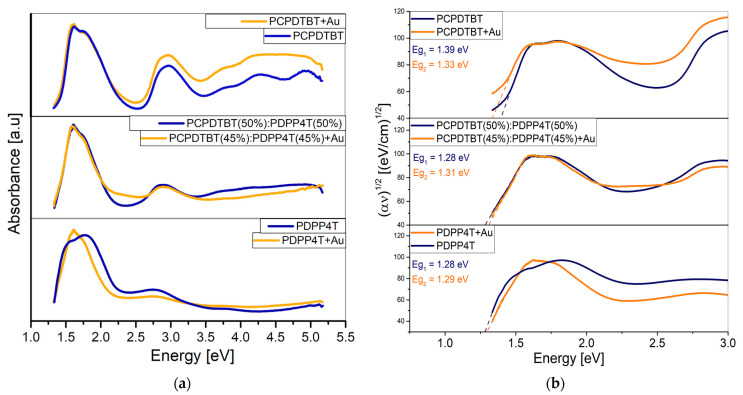
Absorption spectra, determined using Spectra Ray 3 (**a**), and the energy gaps, determined with the Tauc graphical method (**b**).

Figure 2a,b displays the optical absorption spectra and energy band gaps for pure PCPDTBT, PDPP4T, their 1:1 blend, and the corresponding AuNPs (10%) composites for clarity. The remaining absorption spectra are provided in the Supplementary Materials, as shown in Figure S1.

The spectra of pure materials are denoted in dark blue, while the spectra of their composites are shown in dark yellow. In all cases, the first strong absorption band, occurring between 1.25 and 2.25 eV, is associated with π-π* electronic transitions, while the absorption band in the range of 2.25 to 3.5 eV corresponds to n-π* transitions. In the subsequent step, we calculated the absorption coefficient to determine the energy gaps. The absorption coefficient α was obtained using the following relation:

(2) $$\alpha =\frac{\left(2.302\cdot A\right)}{d}$$

Next, using the relationship (*αE*)^(1/2)^ as a function of energy, the energy gap values for each layer were determined through a graphical method by fitting a tangent to the edge of the first absorption band. The results for all samples are presented in Table 4. The increased absorption in the PCPDTBT/Au composite compared to the PCPDTBT spectrum suggests the presence of a plasmonic effect in the material. This is consistent with the observed decrease in the energy gap value. It is well known that the plasmonic effect can simultaneously lead to both a shift in the absorption band and an increase in absorption intensity [36]. This effect arises from changes in the local oscillations of electrons within gold nanoparticles, which influence electronic transition processes in the polymer. AuNPs enhance absorption within specific wavelength ranges, contributing to the overall increase in absorption. In the case of the PDPP4T/Au composite, we observed a slightly lower absorption intensity compared to pure PCPDTBT, along with a narrowing of the absorption band. Additionally, a slight decrease in the energy gap (*E_g_*) value was noted. We attribute this effect to the local passivation of gold nanoparticles [37,38]. We believe this mechanism is due to the interaction of the polymer material with the surface charge of the nanoparticles. The DPP group contains oxygen atoms that bind to AuNPs via dipole attraction. Additionally, this polymer contains long side chains (attached to the DPP group right next to the oxygen atoms) that are electrostatically attracted also to the nanoparticles surface. In this case, long, branched chains present in PDPP4T can physically surround the gold nanoparticles and create a steric barrier that prevents their aggregation and can stabilize them electrostatically.

The minimal changes in the UV-Vis spectrum of the PCPDTBT/PDPP4T/AuNPs composite, compared to the spectrum of the blend, suggest that both the plasmonic effect and passivation occur locally within the prepared samples. We believe that the passivation effect of gold nanoparticles, resulting from the interaction of side chains and oxygen atoms contained in the PDPP4T polymer, quite strongly suppresses the plasmonic effect that appears in the PCPDTBT: AuNPs composite. This is probably caused by the relatively strong screening of nanoparticles by the PDPP4T polymer side chains. However, we believe that the plasmonic effect is still present in the PCPDTBT: PDPP4T: AuNPs composite mixture. This is indicated by the slightly increased optical absorption intensity (Figure 2b) in the region of 2.1–2.45 eV, in the spectrum of the composite compared to the spectrum of the mixture without nanoparticles. Plasmonic bands, in the UV-Vis spectrum, usually appear at values of 520–580 nm wavelength, i.e., 2.1–2.3 eV) [39,40].

### 3.2. Thermal Analysis

**Figure 3:**
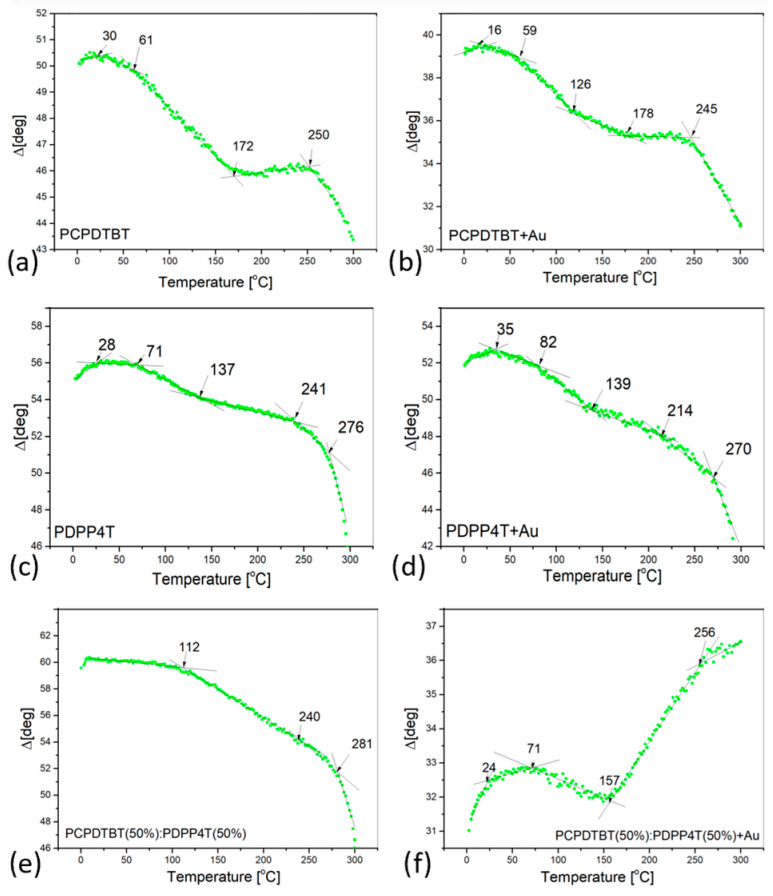
*Δ* at 930 nm as a function of temperature for PCPDTBT (**a**), PDPP4T (**c**), their 50% blend (**e**), and their AuNPs composites with 10% Au (**b**,**d**,**f**).

**Figure 4:**
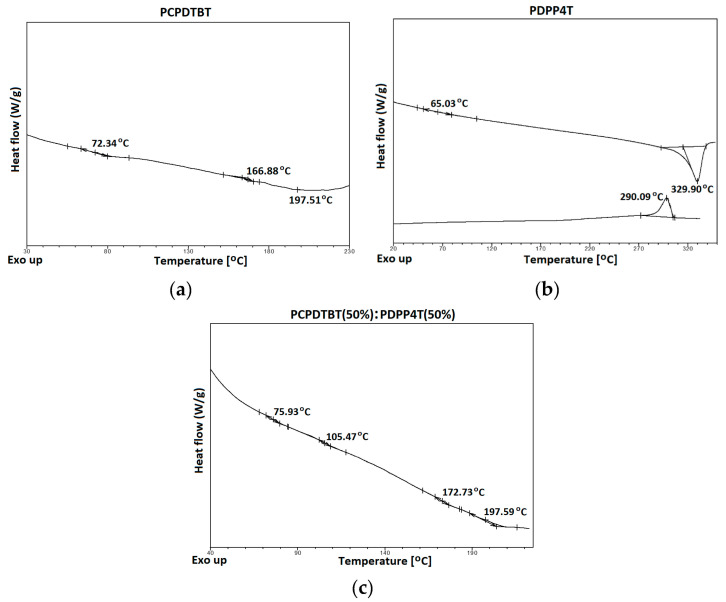
DSC plots, with a heating rate of 20 °C/min, for pure PCPDTBT (**a**) and PDPP4T (**b**) and for PCPDTBT: PDPP4T (1:1) blend (**c**).

Thermal transition analysis was performed using two methods, variable temperature spectroscopic ellipsometry (VTSE) and differential scanning calorimetry (DSC). In previous studies [26,27], we presented ellipsometric angles (*Ψ* or Δ) as a function of temperature for a selected wavelength (λ). For this study, we used λ = 930 nm, which exhibited the lowest dispersion of points. Ellipsometric scans were conducted during heating cycles, with measurements taken every 10 s. The VTSE temperature scans for pure PCPDTBT, PDPP4T, their 1:1 blend, and their AuNPs composites (with a nanoparticle concentration of 10%) are shown in Figure 3a–f. The corresponding DSC curves (Figure 4a–c) were obtained for pure materials and their 1:1 blend. Notably, DSC measurements were not conducted for the AuNPs composites, primarily due to potential nanoparticle aggregation and their size. The small sample volume used for the measurements may not ensure uniform nanoparticle distribution, and the nanoparticles could induce localized temperature variations, disrupting the uniform heat flow within the sample.

Based on the obtained results, it can be concluded that the primary thermal transitions detectable in both the individual materials and their mixtures are characteristic of this type of polymer. These transitions include the glass transition temperature (T_g_), cold crystallization temperature (*T_cc_*), and melting temperature (*T_m_*). For pure materials, the temperatures detected by the DSC and VTSE methods are generally consistent, with any observed differences attributed to several factors. These include the state of the material being tested—where DSC analyzes the powder, while the ellipsometric method examines the prepared film—and differences in the heating rates. In the VTSE method, the heating rate during the temperature cycle is 2 °C/min, while in DSC, it ranges from 10 to 20 °C/min. This manuscript presents results for the most significant materials, i.e., pure polymers, their 1:1 blends, and their composites. Additional thermal data can be found in the Supplementary Materials, specifically in Figure S4a–j. The thermal transitions measured using the VTSE method for pure PCPDTBT and PDPP4T are presented in Figure 3a and Figure 3c, respectively. Based on the DSC results, we have identified several glass transitions in both materials.

For PCPDTBT, the glass transitions were observed around 30 °C, 61 °C, and 172 °C. The corresponding DSC curve revealed *T_g_* values at approximately 72 °C, 167 °C, and 197 °C. Additionally, the cold crystallization temperature (*T_cc_*) observed during the heating cycle at around 250 °C was not detected by DSC. This finding aligns with the literature, which suggests that polymers with a similar chemical structure typically exhibit multiple glass transitions [41]. The first glass transition, occurring around 30 °C, is likely attributed to the DPP group, which is commonly observed around 23 °C in DSC measurements. The two remaining *T_g_* values are likely related to the collective motion of the aromatic rings within the π-conjugated fragments in the polymer’s main chain and the aliphatic side chains. The glass transition at approximately 61 °C is consistent with our earlier findings [26,27,41].

For pure PDPP4T, the glass transition temperatures (*T_g_*) were detected around 28 °C, 71 °C, and 137 °C, with two cold crystallization temperatures (*T_cc_*) observed at approximately 241 °C and 276 °C. The corresponding DSC results showed *T_g_* at around 65 °C and 290 °C. Additionally, the melting point (*T_m_*) was observed around 330 °C. These results are consistent with those from our previous work [41], where we presented the phase diagram for PDPP4T: PDBPyBT for the first time, confirming that the temperatures for pure PDPP4T match those in the phase diagram.

In the 1:1 PCPDTBT: PDPP4T blend, the glass transition was detected around 112 °C, with cold crystallization occurring at around 240 °C and 281 °C. The DSC results revealed additional glass transitions at approximately 75 °C, 105 °C, 172 °C, and 197 °C. This suggests that the internal morphology of the blend film is more uniform compared to the sample prepared for DSC measurement. Notably, the glass transitions observed in the pure materials are not present in the VTSE graph of the mixture.

In the case of PCPDTBT with gold nanoparticles, the glass transitions appeared at about 16, 59, 126, 178 °C. One additional temperature (126 °C) probably originates from local interaction of Au nanoparticles with polymer. As we noted earlier, the presence of gold nanoparticles influences the thermal properties of the material. We believe that gold nanoparticles can weaken interchain interactions in amorphous regions. This can result in higher flexibility of the polymer matrix in the composite compared to the pure material, which results in the occurrence of thermal transitions at lower temperatures. It should be emphasized, however, that these differences are not high. An additional *T_g_* at 126 °C indicates the existence of a second phase in the composite, which could result from the separation of domains with varying gold contents, for instance. This could be the result of areas with varying chain mobility, forming as a result of nanoparticles interacting with particular polymer segments.

In the PDPP4T composite with gold nanoparticles, thermal transitions occur at temperatures of 35, 82, 139, 214, and 270 °C. The last two temperatures are the *T_cc_* temperatures, and the rest are the *T_g_* temperatures. In the case of pure PDPP4T, these temperatures are pronounced at about 28, 71, 137, 241, and 276 °C. Higher *T_g_* values in composite in comparison to pure PDPP4T may indicate that the composite has slightly better crystalline order than the pure material. The lower *T_cc_* value in the case of composite can result from fact that the energy necessary to form the crystalline phase may be decreased if nanoparticles alter the packing of the polymer chains, and the polymer matrix may have a greater ability to form well-developed crystalline regions. In the case of the 1:1 PCPDTBT: PDPP4T mixture, thermal transitions were noticed at about 112, 240, and 281 °C. In the case of the composite of the aforementioned mixture with 10% gold nanoparticles, thermal transitions were present at 24, 71, 157, and 256 °C. We assume that introducing the AuNPs can reduce the mutual compatibility of PCPDTBT and PDPP4T, leading to the formation of separate polymer phases (a higher number of *T_g_* than in the pure material), each with its own glass transition. The separated phases also favor the occurrence of LSPR and the passivation of AuNPs at the same time. The reduction in the glass transition temperature of the polymer matrix in the composite compared to the pure mixture suggests increased crystallinity of the composite.

**Figure 5:**
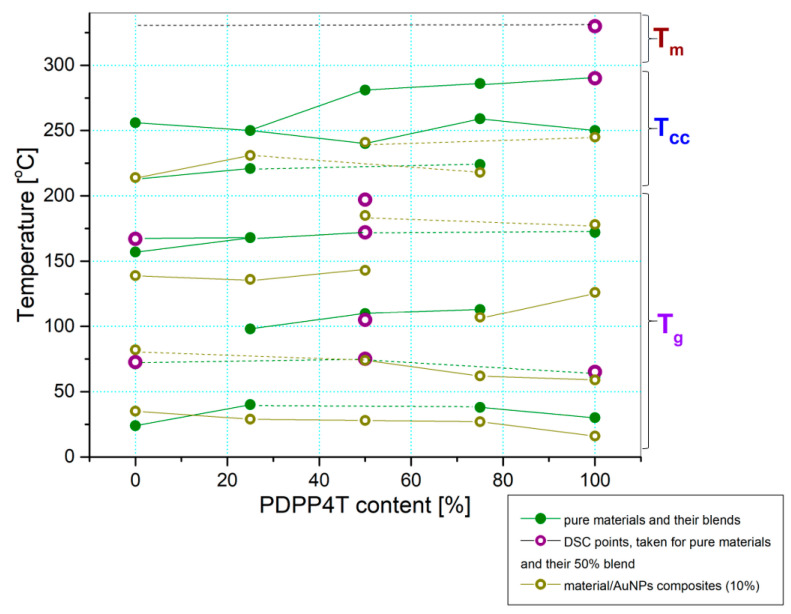
Phase diagram of PCPDTBT, PDPP4T, their PCPDTBT: PDPP4T blend films and their AuNPs composites.

Figure 5 represents the phase diagram, which was constructed using data from DSC and temperature ellipsometry. Thermal transitions are indicated, as follows: light green circles for VTSE measurements, dark green open circles for DSC transitions in pure materials and their blends, and red open circles for VTSE transitions in material/AuNPs composites (10%). The colored regions represent the following specific thermal characteristics: glass transition temperature (*T_g_*) in pink, cold crystallization temperature (*T_cc_*) in blue, and melting temperature (*T_m_*) in orange.

The phase diagram clearly illustrates that thermal transitions differ between pure materials, their blends, and their composites. As previously noted, some transition temperatures are either elevated or reduced. Notably, in the polymer blends, glass transitions occur at approximately 98, 110, and 113 °C for 25%, 50%, and 75% PDPP4T content, respectively. However, these transitions are absent in the composites, suggesting strong interactions between AuNPs and polymer chains.

The most plausible explanation for this phenomenon is a combination of the local plasmonic effect, localized surface plasmon resonance (LSPR) [25,36], and the passivation of AuNPs [37,38]. In the case of the plasmonic effect, nanoparticles influence the glass transition process, rendering the polymer matrix in the composite more fluid over a wider temperature range, thereby diminishing the visibility of the blend’s *T_g_*. Conversely, if passivation dominates, gold nanoparticles stabilize the polymer structure, preventing the distinct glass transition observed in the blends. These findings align well with the optical results, further supporting the proposed mechanisms.

### 3.3. XRD Analysis

**Figure 6:**
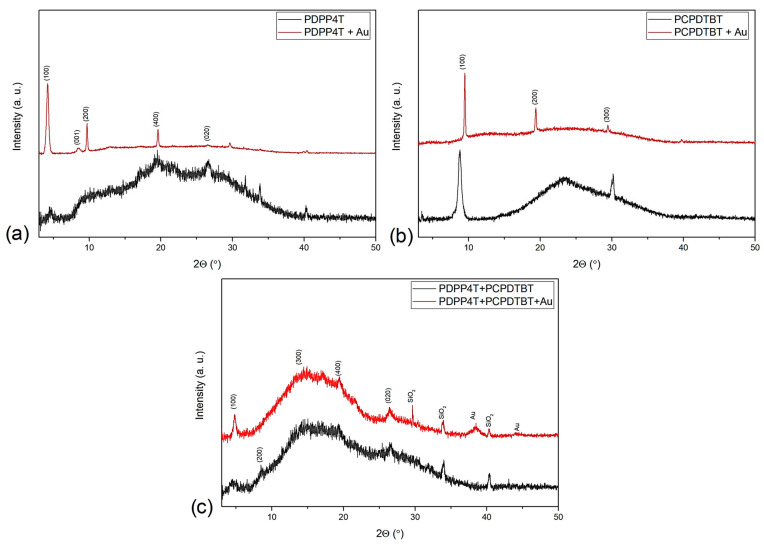
The XRD patterns of PCPDTBT (**a**), PDPP4T (**b**), 1:1 blend (**c**), and their AuNPs composites.

The XRD patterns of pure PCPDTBT and PDPP4T, their 1:1 blend, and the corresponding AuNPs composites are presented in Figure 6a–c; the remaining XRD patterns have been added in the Supplementary Materials in Figure S3. In the case of pure PDPP4T (Figure 6a), a broad amorphous background is observed along with several low-intensity peaks. While the peaks at approximately (100), (400), and (020) exhibit weak intensity in the pure material, their intensity increases in the composite, with additional peaks appearing around (001) and (200). These results indicate that pure PDPP4T consists of a mixed amorphous and crystalline structure. However, in the composite pattern, the presence of additional peaks at (001), (200), and (400), alongside those originating from the pure polymer, suggests that the nanoparticles significantly influence the crystallization process. Moreover, the nanoparticles may form their own crystalline structures, contributing to the diffraction pattern. This suggests the formation of new crystalline domains or strong interactions between the nanoparticles and the polymer matrix. From (Figure 6b), in the spectrum of pure PCPDTBT, two peaks corresponding to crystallization planes appear at approximately (100) and (300). In the PCPDTBT/AuNPs composite spectrum, an additional peak emerges around (200), while the amorphous background is notably reduced. The amorphous background in the pure PCPDTBT pattern suggests that its structure is not highly crystalline. The presence of (100) and (300) peaks indicate a mixed-phase structure, comprising both amorphous and crystalline regions. Comparing this to the composite, the increased intensity and the appearance of the (200) peak suggest a higher degree of crystallinity, likely due to AuNPs acting as nucleation sites around which polymer chains become more ordered [42,43].

This type of pattern indicates an edge-on crystalline orientation, where the crystallization planes are arranged parallel to each other, with each plane positioned perpendicularly to the sample surface.

The XRD patterns of the 1:1 PCPDTBT/PDPP4T blend and its corresponding AuNPs composite are compared in Figure 6c. Both spectra exhibit a broad amorphous background. In the pure blend, weak intensity peaks appear at approximately (100), (200), (400), and (020). However, in the composite, the (100) peak becomes more intense, the (200) peak disappears, the (300) peak emerges, and the intensity of the (400) and (020) peaks remains unchanged. These results indicate that both polymers retain a certain degree of crystallinity. The low-intensity peaks and broad background suggest a predominantly amorphous structure with small regions of ordered crystallization. In the composite, the appearance of the (300) peak suggests the formation of new crystalline structures and interactions between AuNPs and the polymer matrix.

Using Scherrer’s law for the (100) peak, the crystallite sizes were calculated (Table 5). The results show that the crystallite size in PCPDTBT with gold nanoparticles is three times larger than in the pure polymer. In contrast, the crystallization of pure PDPP4T is less affected by the introduction of AuNPs compared to PCPDTBT. For the PCPDTBT/PDPP4T blend, the increase in crystallite size after AuNPs incorporation follows a trend similar to that of pure PCPDTBT, with the crystallite size being three times larger.

### 3.4. Microscopic Analysis

**Figure 7:**
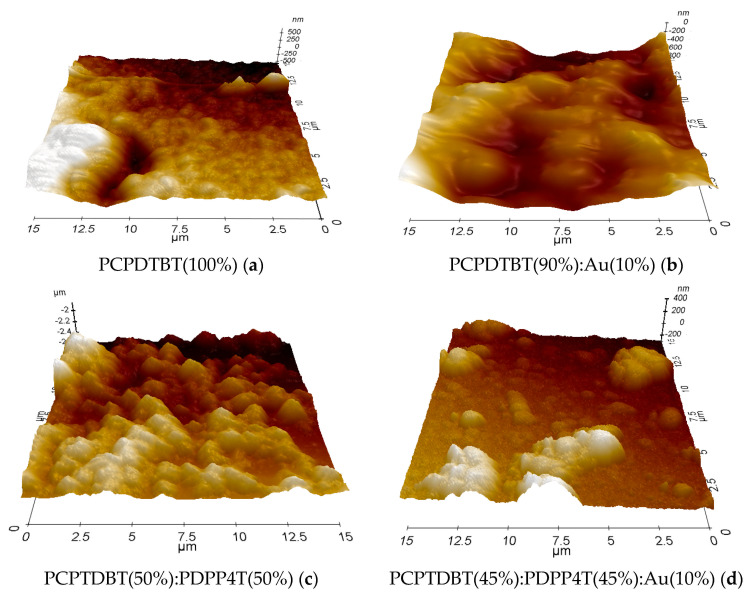

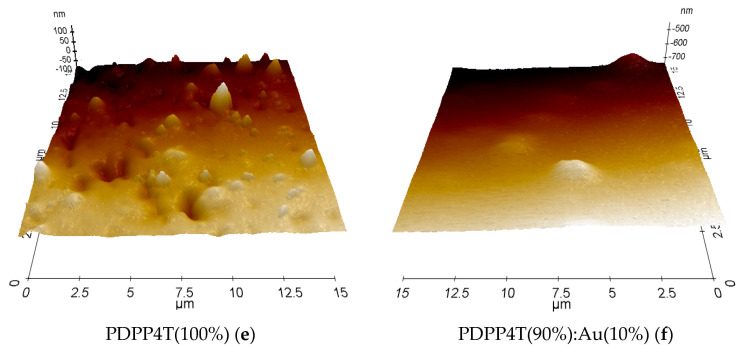
15 × 15 μm 3D topographic surface images of PCPDTBT (**a**), PDPP4T (**c**), their 1:1 blend (**e**), and their corresponding AuNPs composites (**b**,**d**,**f**) obtained using AFM microscope.

Topographic analysis was conducted on layers of pure polymer materials, their blends, and nanocomposite materials containing AuNPs additives. The resulting topographic images are presented in Figure 7. The root mean square roughness (*R_q_*) was used to characterize the surface of all the tested samples.

**Figure 8:**
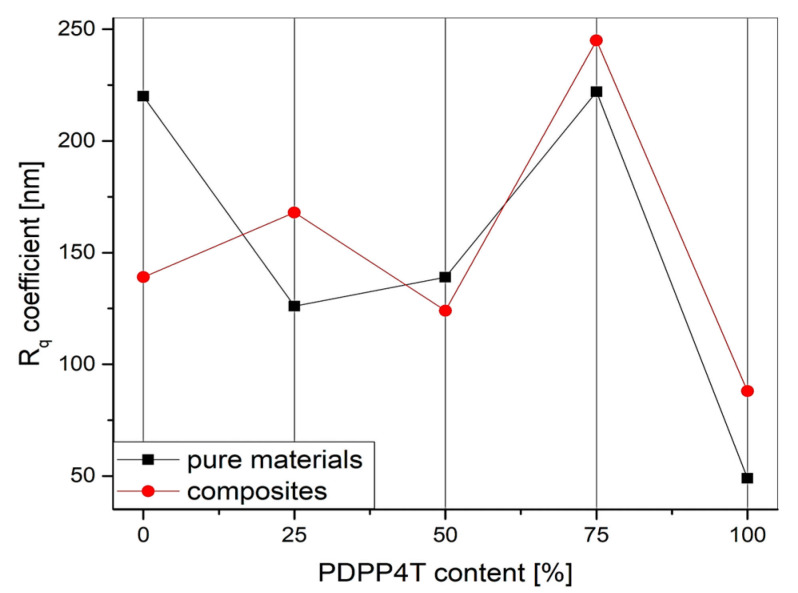
The mean square root of the roughness of PCPDTBT, PDPP4T, their PCPDTBT: PDPP4T blend films and their AuNPs composites.

It is defined with Equation (3) as in [44]:

(3) $$R_{q}=\sqrt{\frac{1}{m}}\sum_{i=1}^{m}{\left(Z_{i}-\bar{Z}\right)}^{2}$$

where *m* is the number of sampled points, *Z_i_* is the height of each point, and $\bar{Z}$ is the mean height value [44]. *R_q_* was determined for three surface sizes, 1 × 1, 2 × 2 and 10 × 10 μm, and is shown in Figure 8.

For the clarity of this manuscript, here, we present the morphology of PCPDTBT, PDPP4T, their 1:1 blend PCPDTBT/PDPP4T, and their corresponding composites. The rest of the AFM morphology pictures are presented in the Supplementary Materials in Figure S2a–d).

The results of the study suggest that the pure PCPDTBT polymer thin film (Figure 7a) has a relatively homogeneous surface, with clearly visible loss and agglomeration. However, water defects on the surface of the thin film are most likely caused by the agglomeration of the polymer material caused by the volatility of solvent and interactions of polymer solvent pair [45]. The remaining surface of the thin film remains smooth, which is characteristic of PCPDTBT layers resulting from solid state ordering induced by racemic side chains [46]. The addition of AuNPs caused the development of the film surface, which, however, remained continuous and relatively smooth. The results shown in (Figure 7b) indicate that the addition of Au nanoparticles induced microstructural changes, likely due to agglomeration occurring during the mixing of different components in the film [47].

Thin films of blended materials (Figure 7c) exhibit a homogeneous surface with distinct crystalline structures. Small agglomerated polymer formations are visible; however, the absence of larger agglomerates (above 2 µm) suggests that blending promotes the ordering of polymer chains. However, considering the findings of other researchers, such as Wang et al. [48], it is expected that pure polymer semiconductor layers generally have lower surface roughness compared to blended films. For the blended film (Figure 7d), the addition of gold nanoparticles led to a reduction in surface roughness. This effect may be attributed to the influence of the nanoadditive on the ordering of polymer chains or a phenomenon observed by Nathanael et al. [49]. In their study, an increase in TiO₂ nanoparticle concentration enhanced the uniformity of the HAp/TiO₂ film, while reducing its roughness. Similarly, research by Predoi et al. [50] demonstrated that decreased composite layer roughness resulted from the formation of uniformly distributed nanoconglomerates on the surface.

The pure PDPP4T films (Figure 7e) exhibit a more homogeneous surface compared to the pure PCPDTBT layers, with no material agglomerates larger than 2 µm. Crystalline fractions are visible on the film’s surface, a characteristic feature of this polymer due to the crystallization tendency of its alkyl chains attached to the DPP unit. These chains act as high-solubilizing groups, promoting tight molecular packing in the film [51]. Similar to PCPDTBT and blend thin films, the incorporation of AuNPs results in a smoother surface. In the case of PDPP4T/AuNPs layers (Figure 7f), this smoothing effect may be attributed to the formation of Au nanoclusters within the layers or the development of crystalline or semi-crystalline structures.

The surface topography of the produced layers was quantitatively analyzed by determining the surface roughness coefficient (*R_q_*). The addition of Au nanoadditives to both pure PCPDTBT and its blend resulted in a reduction in surface roughness. This effect was more pronounced in pure PCPDTBT, likely due to the presence of larger agglomerates in the pristine material. Interestingly, for PDPP4T films, the *R_q_* coefficient increased after incorporating the nanoadditive, despite the qualitatively smoother surface observed in microscopic analysis. However, given that the initial roughness of pure PDPP4T is significantly lower than that of PCPDTBT, it can be inferred that the formation of Au nanoclusters contributed to the increase in roughness. Furthermore, the results suggest that the *R_q_* coefficient of the blended film indicates the effective mixing of the materials, supporting the hypothesis of improved uniformity in the blend structure.

### 3.5. General Discussion

**Scheme 2:**
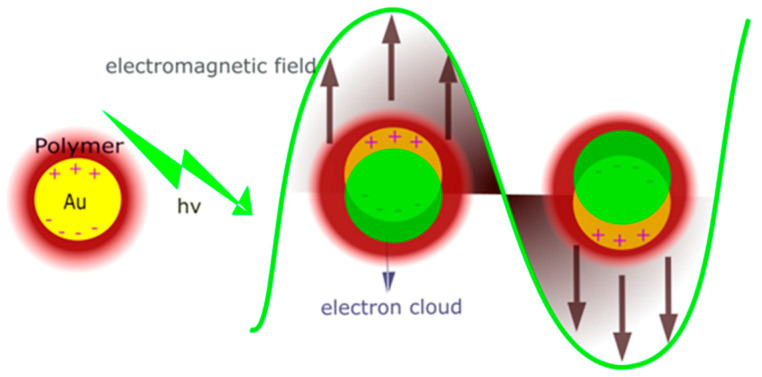
Representation of the localized surface plasmonic effect in the polymer/AuNPs composite.

The results obtained for pure materials and their composites indicate that several phenomena occur simultaneously in the tested samples. These include the plasmonic effect, the passivation of gold nanoparticles, and the role of AuNPs as nucleation centers influencing polymer crystallization. The plasmonic effect is most pronounced in the PCPDTBT/Au composite. A comparison of its optical absorption spectrum with that of the pure material reveals that the gold nanoparticles significantly alter the electronic structure. This corresponds to a localized plasmonic effect, where surface plasmons are excited directly on zero-dimensional AuNPs. These plasmons resonate around the nanoparticles, as illustrated in Scheme 2. It is important to note that the plasmonic effect is primarily associated with AuNPs that are well-isolated within the polymer matrix [52,53].

We noticed that the plasmonic effect also indirectly influences the thermal transitions of the polymer. The most evident example is the lower cold crystallization temperature (*T_cc_*) in the PCPDTBT/Au composite, where *T_cc_* decreases to 214 °C compared to 256 °C in pure PCPDTBT. These changes in thermal transitions arise from the ability of AuNPs to absorb light, generating localized heating through localized surface plasmon resonance (LSPR). This phenomenon enhances polymer chain mobility, accelerating the crystallization process. This effect is clearly reflected in the XRD spectra, where an increase in the crystalline peak intensity is observed.

For PDPP4T, the addition of gold nanoparticles leads to a noticeable reconstruction of the polymer’s crystal structure and improved structural stability. The results suggest that AuNPs promote the formation of lamellae, which align in parallel planes perpendicular to the sample substrate. As previously mentioned, this corresponds to an edge-on structure, which facilitates charge transport via π-stacking interactions. In the case of the PCPDTBT/PDPP4T blend, the internal structure remains predominantly amorphous, with localized crystalline phases. X-ray diffraction results confirm this, showing a strong amorphous background alongside weak peaks corresponding to crystalline planes. Additionally, thermal and microscopic analyses indicate a high degree of miscibility between the two polymers. The characteristic thermal transitions of pure PCPDTBT and PDPP4T phases are nearly absent in the 1:1 blend, suggesting effective mixing at the molecular level. For the corresponding PCPDTBT/PDPP4T/Au composite, the emergence of additional crystalline peaks in the XRD pattern suggests the formation of new crystalline domains or strong interactions between the nanoparticles and the polymer matrix. This indicates that both plasmonic and passivation effects are present in the composite, but to a lesser extent than in the Au nanocomposites of the individual polymers.

The presence of this crystalline arrangement indicates the potential application of PDPP4T/Au composites as active materials in organic field-effect transistors (OFETs).

## 4. Conclusions

In this study, we investigated the thermal and physical properties of a novel polymer blend—PCPDTBT: PDPP4T—as well as its composite with gold nanoparticles (AuNPs). Our findings indicate that the local plasmonic effect was not uniformly present in all samples but was most pronounced in the PCPDTBT/Au composite. This was evidenced by a significant reconstruction of the electronic band structure observed in the UV-Vis spectra.

AuNPs had a pronounced influence on the thermal transitions within the polymer matrix. Notable effects included a significant decrease in the cold crystallization temperature (*T_cc_*) of PCPDTBT and the disappearance of the glass transition temperature associated with the PCPDTBT/PDPP4T blend phase at different PDPP4T concentrations in the composites. In the case of the PCPDTBT: PDPP4T (1:1) mixture layer and its composite with AuNPs, the obtained thermal test results indicate the high miscibility of the tested polymers. Changes in thermal transitions, visible in the prepared phase diagram, suggest that, in the case of the composite, the separated phases promote the occurrence of passivation of gold nanoparticles and the plasmonic effect at the same time, which is strongly reflected in the optical absorption spectra also.

Furthermore, the incorporation of AuNPs had a notable impact on the internal structure of the tested layers, particularly their crystallinity. The most significant structural changes were observed in the PDPP4T/Au composite, where gold nanoparticles acted as nucleation centers, what is pronounced with the bigger, average size of appeared crystallites. Through interactions with the polymer chains, the AuNPs promoted a higher degree of molecular ordering compared to the pure polymer, leading to the formation of an edge-on crystalline orientation. For the PCPDTBT/PDPP4T composite, we identified the simultaneous occurrence of two key phenomena: a plasmonic effect—though weaker than that observed in the PCPDTBT/Au composite—and enhanced structural ordering. This was confirmed by the presence of additional peaks in the XRD spectra, indicating the formation of new crystalline domains.

In conclusion, the observed surface plasmon resonance and passivation effects induced by the presence of AuNPs in these materials presents exciting prospects for future research. Further investigations should focus on determining whether these phenomena can enhance the quantum efficiency of the investigated materials, opening up new possibilities for their application in optoelectronic devices.

## Figures and Tables

**Table 1:** The individual concentrations of pure materials and their blends.

Sample No	1	2	3	4	5
PCPDTBT [%]	100	75	50	25	0
PDPP4T [%]	0	25	50	75	100

**Table 2:** The individual concentrations of the composites of polymers and their blends.

Sample No	6	7	8	9	10
PCPDTBT [%]	90	70	45	20	0
PDPP4T [%]	0	20	45	70	90
Au [%]	10	10	10	10	10

**Table 3:** The individual thickness values of prepared samples using spin coating, deposited onto silicon and microscopic glass substrates (number of samples are the same with Table 1 and Table 2).

		1	2	3	4	5	6	7	8	9	10
d [nm]	SiO_2_	111	113	114	93	110	90	100	116	111	97
	glass	416	511	480	473	490	503	489	470	501	490

**Table 4:** Band gap values for PCPDTBT, PDPP4T, their blends, and nanocomposites with Au nanoparticles.

PCPDTBT [%]	PDPP4T [%]	Eg [eV]	Eg [eV] (+Au)
100	0	1.39	1.33
75	25	1.32	1.38
50	50	1.28	1.31
25	75	1.22	1.29
0	100	1.28	1.29

**Table 5:** The size of crystallites calculated for pure materials and their composites.

Sample	Crystal Size (nm)
PCPDTBT	16.1
+Au	47.3
PDPP4T	18.0
+Au	31.6
BLEND	21.5
+Au	60.3

## Data Availability

Data are contained within the article and Supplementary Materials.

## Supplementary Materials

The following supporting information can be downloaded at: https://www.mdpi.com/article/10.3390/polym17050704/s1, Figure S1: Absorption spectra of pure PCPDTBT, PDPP4T, their blends, AuNPs composites (a) and their energy band gaps (b), Figure S2: 15 × 15 μm 3D topographic surface images of PCPTDBT(75%):PDPP4T(25%) (a) PCPTDBT(70%):PDPP4T(20%):Au(10%) (b) PCPTDBT(25%):PDPP4T(75%) (c) and PCPTDBT(20%):PDPP4T(70%):Au(10%) (d), Figure S3: XRD patterns of PCPDTBT, PDPP4T, their blends and AuNPs composites, Figure S4: Ellipsometric Ψ i ∆ temperature scans at wavelength λ = 930 nm: (a) PCPDTBT(100%), (b) PCPDTBT(90%) + Au(10%), (c) PCPDTBT(75%):PDPP4T(25%), (d) PCPDTBT(70%):PDPP4T(20%) + Au(10%), (e) PCPDTBT(50%):PDPP4T(50%), (f) PCPDTBT(45%):PDPP4T(45%) + Au(10%), (g) PCPDTBT(25%):PDPP4T(75%), (h) PCPDTBT(20%):PDPP4T(70%) + Au(10%), (i) PDPP4T(100%), (j) PDPP4T(90%) + Au(10%)
