# Supplementary material for: Investigations on Thermal Transitions in PDPP4T/PCPDTBT/AuNPs Composite Films Using Variable Temperature Ellipsometry
Source: Polymers (Basel). 2025 Mar 6;17(5):704. doi: 10.3390/polym17050704 (PMC11902429; doi:10.3390/polym17050704)
Supplement: Supplementary file 1 [file polymers-17-00704-s001.zip › polymers-3482043-supplementary.pdf]

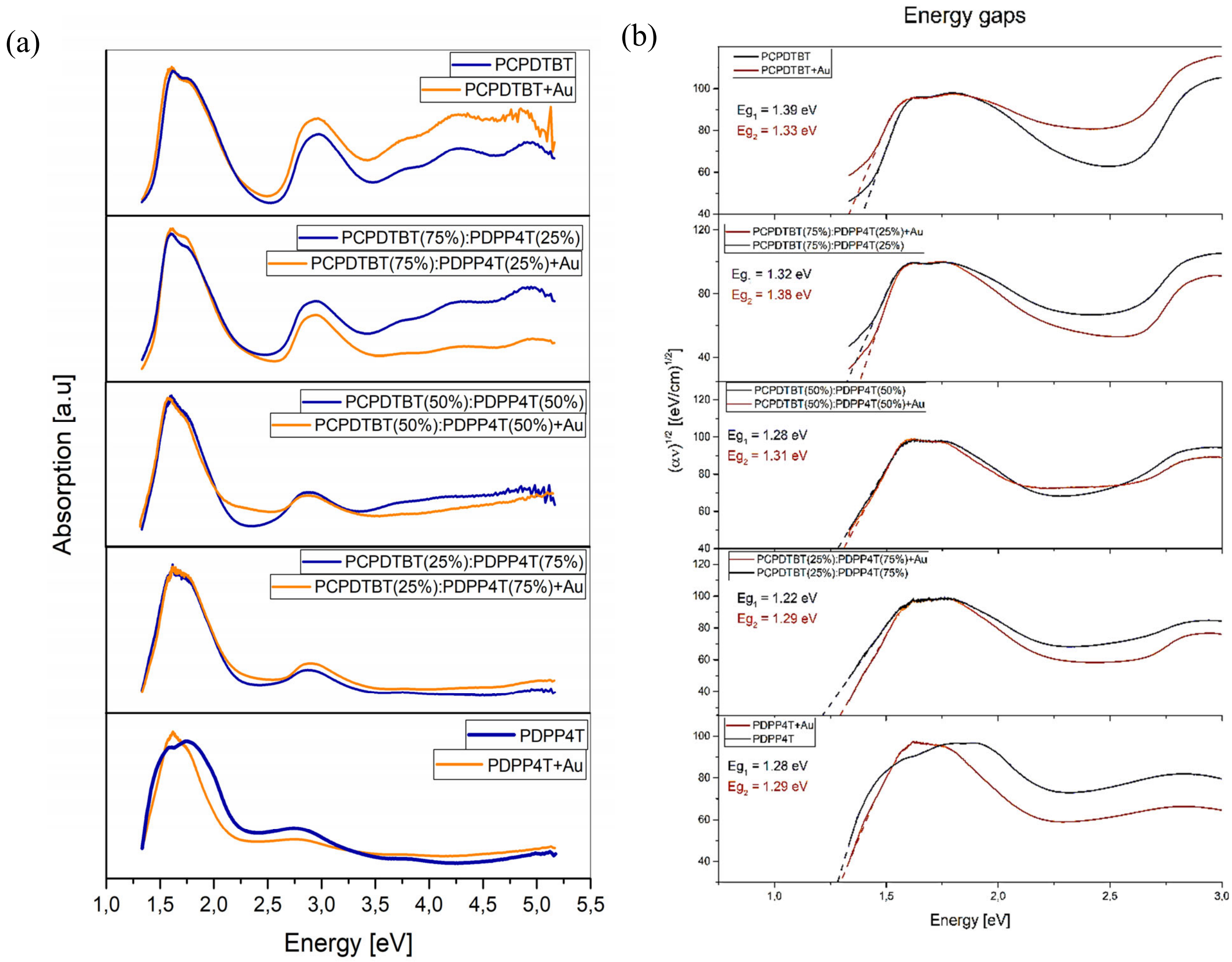

Figure S1. Absorption spectra of pure PCPDTBT, PDPP4T, their blends, AuNPs composites (a) and their energy band gaps (b).

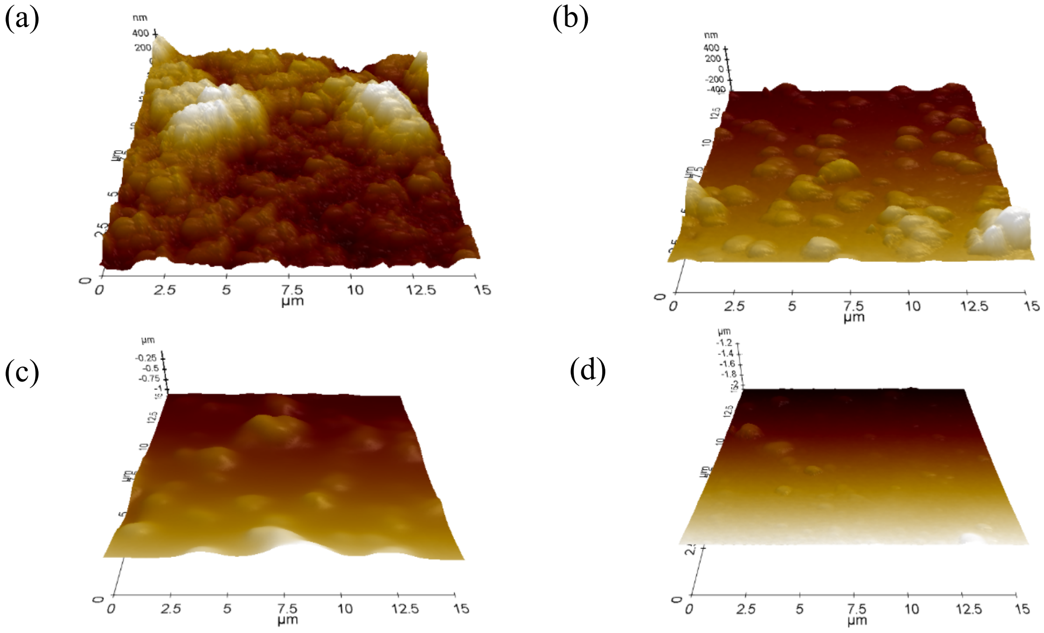

Figure S2. 15 × 15 µm 3D topographic surface images of PCPTDBT(75%):PDPP4T(25%) (a) PCPTDBT(70%):PDPP4T(20%):Au(10%) (b) PCPTDBT(25%):PDPP4T(75%)(c) and PCPTDBT(20%):PDPP4T(70%):Au(10%) (d).

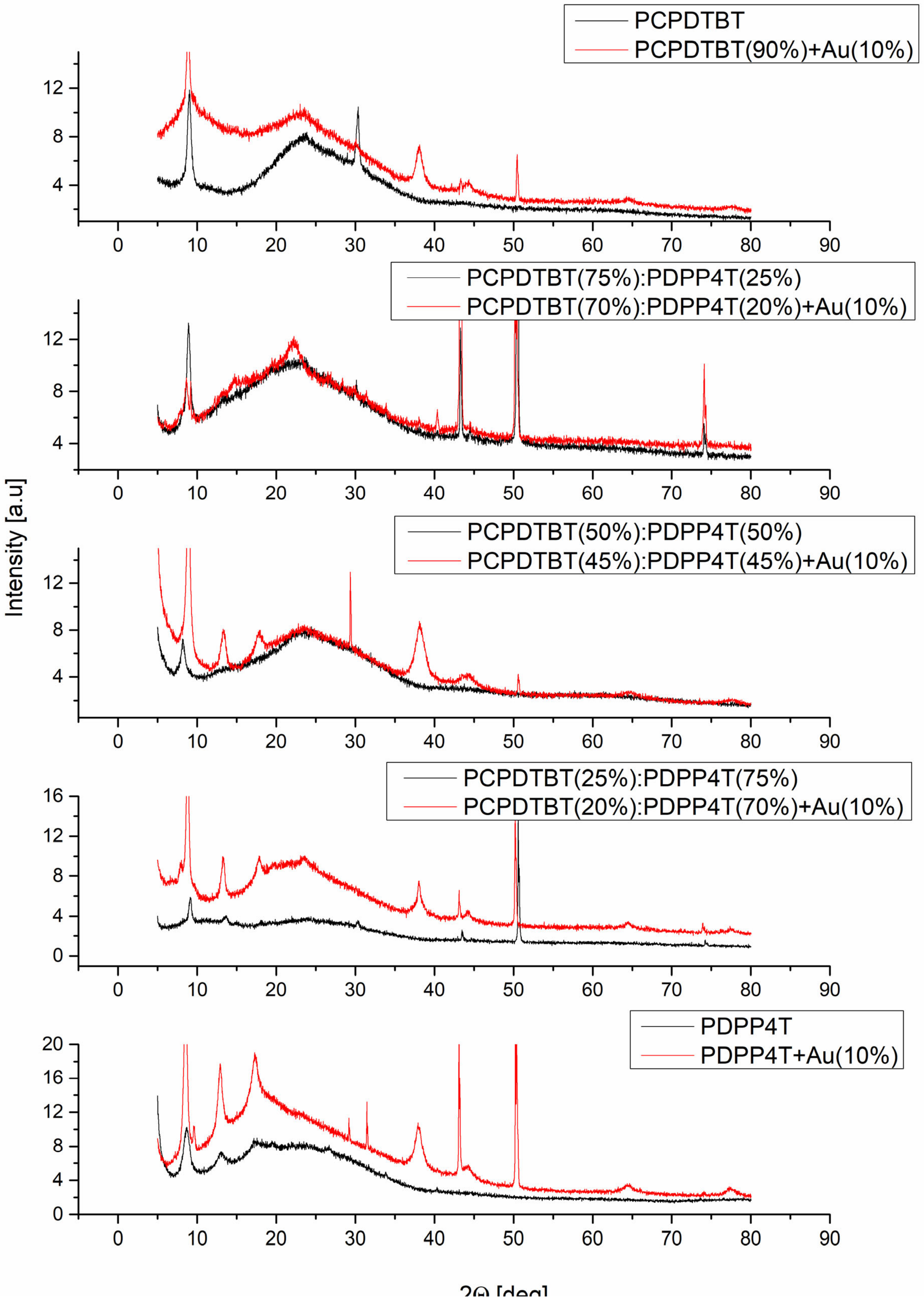

Figure S3. XRD patterns of PCPDTBT, PDPP4T, their blends and AuNPs composites.

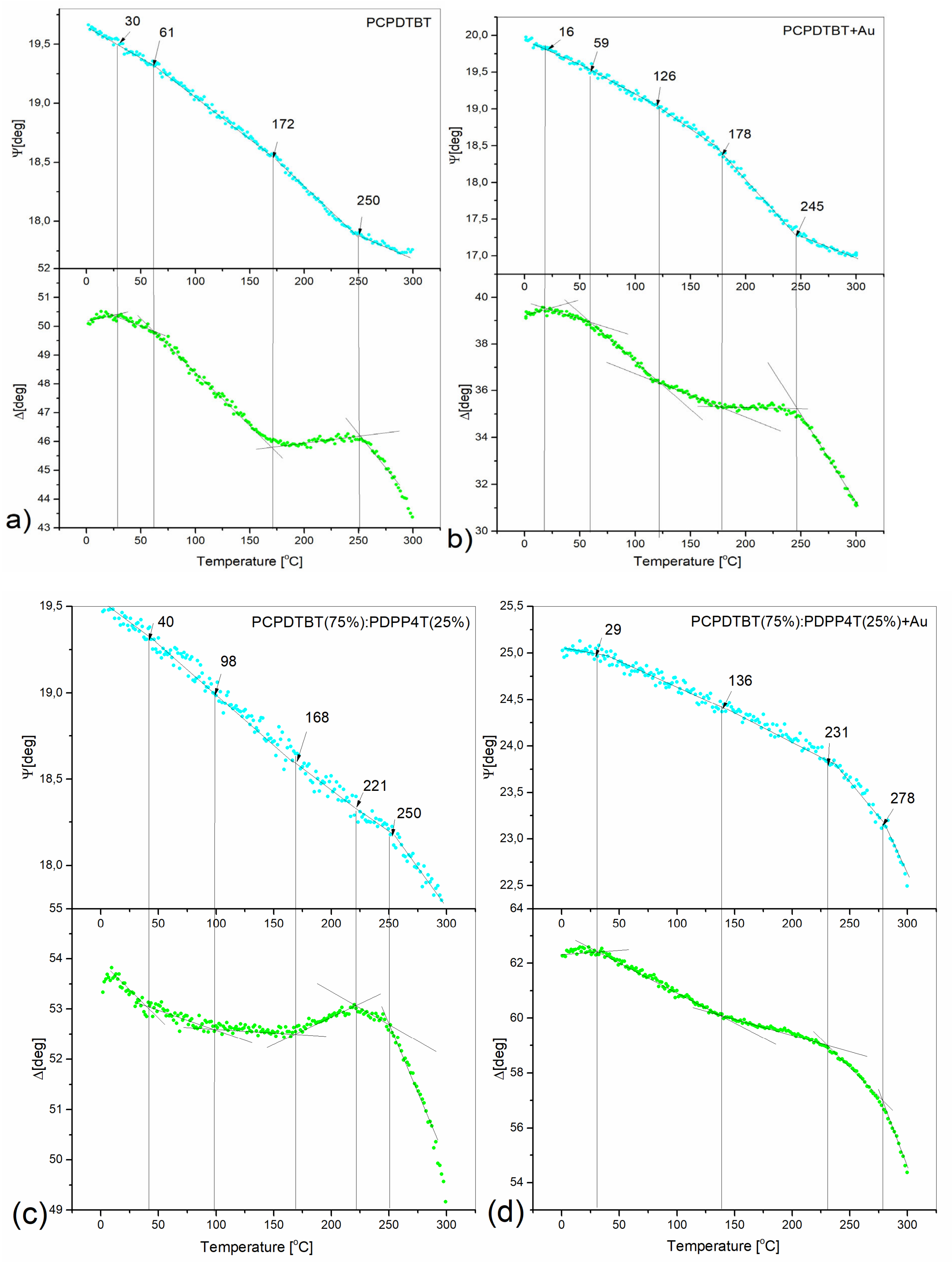

PCPDTBT
30
61
172
250
Ψ[deg]
Δ[deg]
a)
Temperature [°C]
PCPDTBT+Au
16
59
126
178
245
Ψ[deg]
Δ[deg]
b)
Temperature [°C]
PCPDTBT(75%):PDPP4T(25%)
40
98
168
221
250
Ψ[deg]
Δ[deg]
(c)
Temperature [°C]
PCPDTBT(75%):PDPP4T(25%)+Au
29
136
231
278
Ψ[deg]
Δ[deg]
(d)
Temperature [°C]

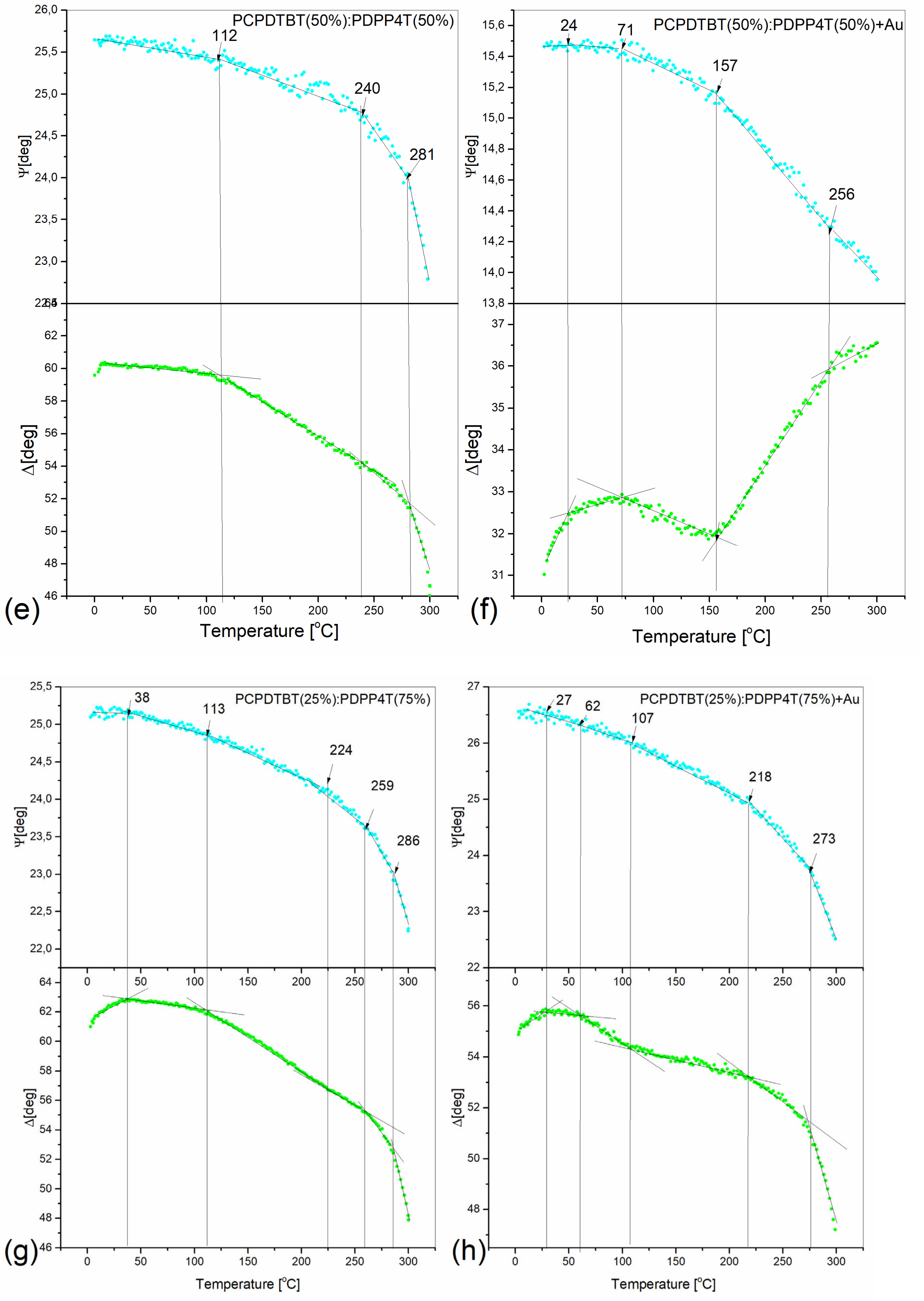

PCPDTBT(50%):PDPP4T(50%)
112
240
281
Ψ[deg]
Δ[deg]
(e)
Temperature [°C]
PCPDTBT(50%):PDPP4T(50%)+Au
24
71
157
256
Ψ[deg]
Δ[deg]
(f)
Temperature [°C]
PCPDTBT(25%):PDPP4T(75%)
38
113
224
259
286
Ψ[deg]
Δ[deg]
(g)
Temperature [°C]
PCPDTBT(25%):PDPP4T(75%)+Au
27
62
107
218
273
Ψ[deg]
Δ[deg]
(h)
Temperature [°C]

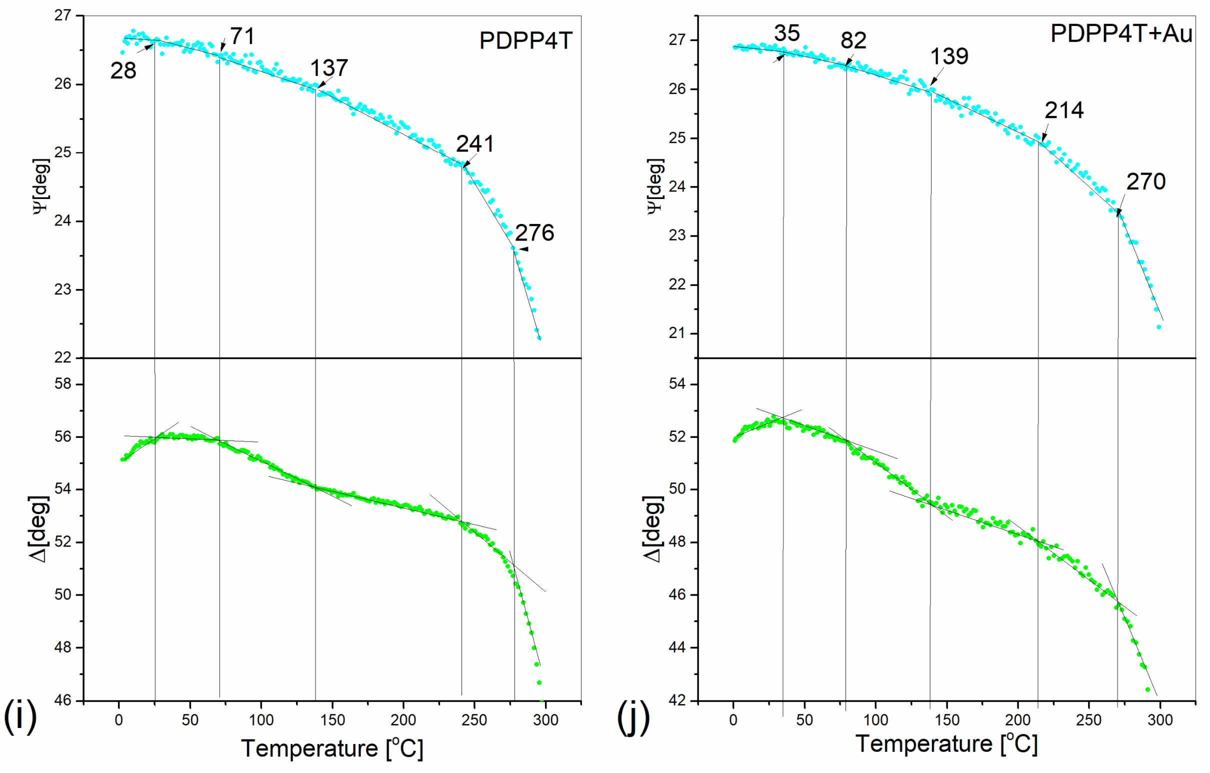

Figure S4. Ellipsometric Ψ i Δ temperature scans at wavelength λ = 930 nm. (a) PCPDTBT(100%), (b) PCPDTBT(90%)+Au(10%), (c) PCPDTBT(75%):PDPP4T(25%), (d) PCPDTBT(70%):PDPP4T(20%)+Au(10%), (e) PCPDTBT(50%):PDPP4T(50%), (f) PCPDTBT(45%):PDPP4T(45%)+Au(10%), (g) PCPDTBT(25%):PDPP4T(75%), (h) PCPDTBT(20%):PDPP4T(70%)+Au(10%), (i) PDPP4T(100%), (j) PDPP4T(90%)+Au(10%).
